# Early cognizance of folic acid supplementation among pregnant women in the prevention of cleft lip and palate- a questionnaire study

**DOI:** 10.1016/j.jobcr.2025.01.021

**Published:** 2025-02-24

**Authors:** Komagan Prabhu Nakkeeran, Akshata Jitender, Selvakumar Thulasiraman, Velavan Krishnan, Muthalagappan P.L.

**Affiliations:** SRM Dental College, Ramapuram Campus Chennai, Tamil Nadu, India

**Keywords:** folic acid, Folate, Neural tube defects, Prevention, Genetics of neural tube defects, Pregnancy supplementation

## Abstract

**Background:**

The awareness of the symbiotic correlation between folic acid and its role in preventing orofacial clefts in pregnant mothers needs to be established during the early gestational period, proving that consumption in both dietary and supplementation forms has positive effects on the mother and the developing fetus.

**Aim:**

The present study raises awareness of the benefits and use of folic acid supplementation in the early phases of gestation.

**Materials and methods:**

A questionnaire survey was conducted over 9 months. It was designed with a total of 13 queries, and a sizable sample of 100 women were personally interviewed after obtaining verbal consent.

**Results:**

A total of 100 pregnant women of different age groups participated in the questionnaire study. Of these, only 11 of the respondents were found to be aware of the benefits of folic acid as a nutritional supplement, and 29 respondents were on folic acid supplementation. Almost 52 percent of those surveyed failed to take folate supplementation in the first two trimesters.

**Conclusion:**

The survey highlights the urgent need for information access and awareness to understand the benefits of supplementary folic acid among pregnant mothers in the prevention of orofacial clefts and overall well-being.

## Graphical abstract

**Figure:**
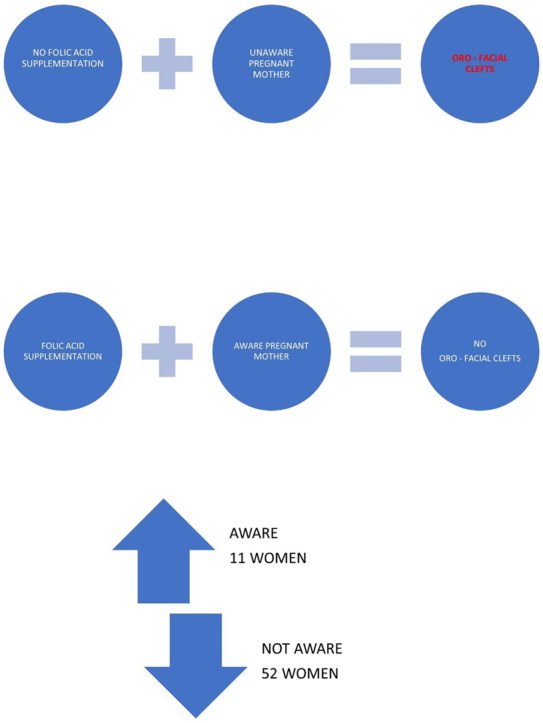
Conclusive graphical overview.

## 1 Introduction

Orofacial clefts are one of the most commonly reported birth defects among infants worldwide. Cleft lip and palate occur in approximately one in 700 live births.^1^. According to ICMR – AIIMS 35,000 new cases are recognized every year in India. Orofacial clefts are caused by the non-fusion of lips with the palate resulting in a split or opening in the upper lip and palate during the development of the unborn in the womb. This is known to arise between 6 and 9 weeks of pregnancy.^2^

Research indicates that adequate intake of folic acid before conception and during early pregnancy can significantly decrease the occurrence of these birth defects. Healthcare providers often recommend prenatal vitamins containing folic acid to expectant mothers as a preventive measure.^3^ Ensuring sufficient folic acid intake is a fundamental component of prenatal care and can contribute to the healthy development of the fetus. It has long been known to minimize neural tube defects (NTDs). Folic acid augmentation has been identified as a crucial factor in reducing the risk of orofacial clefts during pregnancy.

In pregnancy the demand for folate increases for both the mother and the fetus, hence the consumption of food containing B_9_ along with the required supplementation is vital. Folic acid/folate is naturally present in a wide variety of foods, including vegetables (especially dark green leafy vegetables), fruits and fruit juices, nuts, beans, peas, seafood, eggs, dairy products, meat, poultry, and grains.^4^ Spinach, liver, asparagus, and Brussels sprouts are among the foods with the highest folate levels. The nutritional intake of the mother often depends on the socio-economic status of the family. As sufficient dietary consumption cannot be ensured at all times, appropriate use of folic acid supplementation in pregnancy should be made mandatory.

RBC folate levels are useful indexes in assessing the duration of deficiency.^5^^,^^6^

Given the lack of awareness of dietary sources and the overall prevalence of folic acid deficiency, as reported in a recent study, at 11 %, conducted in an urban community of apparently healthy adults from the southern part of India.^7^

The objective of the present questionnaire survey is to elicit awareness about folic acid, its use, and the estimated benefits of vitamin B_9_ as dietary intake and supplementation among pregnant women, particularly about the orofacial clefts.

In pregnant mothers, the amount of folic acid absorbed by the fetus is greater, hence the consumption of food containing B_9_ along with the required supplementation is vital. The lack of awareness of dietary sources and the overall prevalence of folic acid deficiency of 11.1 % was reported in a recent study conducted in an urban community of apparently healthy adults from the southern part of India.^7^

During pregnancy, a prenatal vitamin is taken each day that has 600 mcg of folic acid in it. Folic acid only works to prevent neural tube defects (NTD) before and during the first few weeks of pregnancy.

RBC folate levels are useful indexes in assessing the duration of deficiency.^5^,^6^

The nutritional intake of the mother often depends on the socio-economic status of the family. Folic acid/folate is naturally present in a wide variety of foods, including vegetables (especially dark green leafy vegetables), fruits and fruit juices, nuts, beans, peas, seafood, eggs, dairy products, meat, poultry, and grains.^5^ Spinach, liver, asparagus, and Brussels sprouts are among the foods with the highest folate levels.

The objective of the present questionnaire survey is to elicit awareness of folic acid use and the estimated benefits of vitamin B_9_ as dietary intake and supplementation among pregnant women.

## 2 Materials and methods

The questionnaire survey was completed over 9 months (from August 2023–April 2024) on pregnant women reporting to the SRM General Hospital as outpatients. They were personally interviewed following an informed verbal consent.

**Study design:** A study of the purposive sampling method was designed to analyze the awareness and benefits of folic acid consumption among the Indian population.

**Subject population:** The subjects selected for conducting the survey comprised pregnant women attending the outpatient departments of SRM General Hospital, Chennai. Their educational and income level was not associated with Folic Acid knowledge.

**Participants Questionnaire:** The designed survey contained 13 questions relating to the perception of the essentiality of folic acid in gravid women. This included age, time of pregnancy, number of children, time of visit to the gynecologist, previous child being born with cleft lip or cleft palate anomalies, conceiving development of any health-related issues, any medications or vaccinations taken at the time of conception, duration of medication, regular hospital visits, age of conceiving, any medications being taken since the time of conception, marriage a consanguineous or non-consanguineous one and awareness of folic acid in prevention of developmental anomalies.

## 3 Results

A total of 100 participants answered the questionnaire. The socio-demographic data and the percentage (see Table 1).

**Table 1:** Percentages of sociodemographic variables

SOCIODEMOGRAPHIC VARIABLES		N %
**NUMBER OF CHILDREN**	1^ST^ CHILD 2^ND^ CHILD 3^RD^ CHILD	59 % 37 % 4 %
**TRIMESTERS**	I	37 (37 %)
	II	39 (39 %)
	III	24 (24 %)
**MARRIAGE**	CONSANGUINEOUS	44 %
	NON - CONSANGUINEOUS	56 %
**MEDICATION SINCETHE TIME OF CONCEPTION (FOLIC ACID)**	FOLIC ACID	29 %
	NO MEDICATION	71 %
		
		
**BENEFITS OF FOLIC ACID**	YES	11 %
	NO	89 %
**OTHER** **MULTIVITAMINS**	CALCIUM CALCIUM AND VITAMIN	8 % 2 % 9 % 4 % 1 % 1 % 8 % 2 % 65 %
	IRON	
	IRON AND CALCIUM	
	RANTAC	
	SHELCAL AND LIVOGEN	
	VITAMIN	
	VITAMIN AND IRON	
	NONE	

The distribution of questions and scores related to knowledge and awareness indicated that 89 % of the 100 participants did not know about the nutritional significance of folic acid, while 11 % were informed about its benefits. The source of data related to the awareness of folic acid was first-hand data given by the participants.

A cumulative of 76 amongst the 100 were grouped under the 1st and 2nd trimesters, the former being 37 and the latter accounting for 39. The first and second trimesters were taken as an important reference to elicit the role of folic acid and essential multivitamins vital for embryogenesis.

Of the 100 respondents, 12 had folic acid medications in the 1st trimester, 17 in the 2nd trimester, and 5 in the 3rd trimester which showed the lack of folic acid as an adjuvant medication to be prescribed during their routine prenatal checkup. Most among the pregnant group, accounting for 71 did not know the role of nutritional supplements in preventing developmental anomalies like cleft lip and palate. Almost 52 percent of the respondents were not taking folic acid in the first two trimesters (Table 2).

**Figure:**
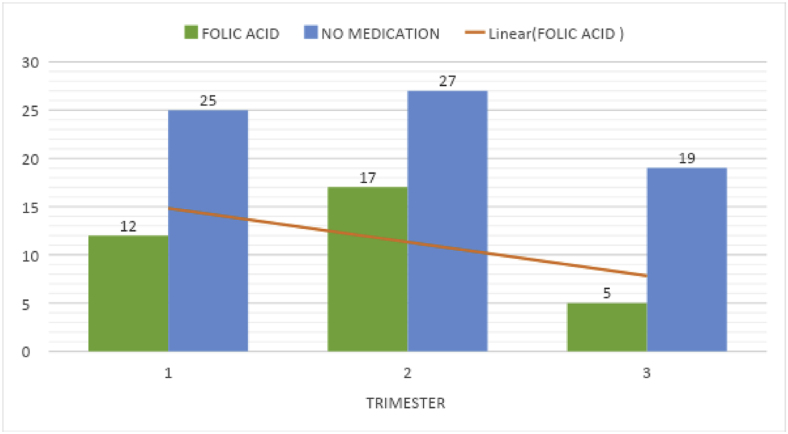

**Table 2:** Correlation between folic acid and trimester.

		Medication since the time of conception (folic acid)		Total	Chi-square value	p-value
		folic acid	no med			
Pregnancy time (trimester)	1	12	25	37	1.048	0.592
	2	15	27	39		
	3	5	19	24		
Total		34	71	100		

This correlation is not statistically significant (see Table 3).

**Table 3:** Correlation of gestational time of visit and benefits of folic acid.

		benefits of folic acid		Total	Chi-square value	p-value
		no	yes			
Time of visit (weeks)	3	3	0	3	32.165	**.000**
	4	11	1	12		
	5	0	2	2		
	6	16	5	21		
	7	3	0	3		
	8	45	2	47		
	12	11	0	11		
	16	0	1	1		
Total		89	11	100		

Even among seasoned mothers with multiple children, the intake of folic acid remains low compared to first-time pregnant women. An analysis across all trimesters reveals that the number of pregnant women taking folic acid decreases as the number of children increases. The highest intake occurs during the first pregnancy, while those with more than three children show the least consumption.

Regarding pregnant women who are taking vitamin supplements alongside folic acid, 19 are first-time mothers, 17 are expecting their second child, and only 1 mother seeking care for her third child was prescribed folic acid along with other multivitamins. This suggests a significant lack of awareness about folic acid among mothers who are already having a second child.

When examining the relationship between the understanding of folic acid's benefits and its consumption, only 11 women reported being informed about its advantages, with 8 being positively engaged in both knowing and consuming folic acid, while 3 were aware but did not take the supplementation.

The timing of prenatal visits and awareness of folic acid's benefits highlight a need for better education. In this study, only three mothers took folic acid between 3 and 5 weeks, with usage peaking at 6–8 weeks but dropping significantly by 12–16 weeks; during this period, most mothers were not taking it. This suggests a concerning lack of awareness about folic acid's importance during crucial weeks despite the necessity for consistent intake. The findings revealed a statistically significant correlation between visit timing and understanding of folic acid's benefits.

The age bracket of 17–20 years comprises 23 %, the lowest number of mothers taking the folic acid supplementation, revealing a lack of knowledge in younger age groups.

The highest folic acid consumption was in the age group of 21–25 with 42 % followed by the 26–30-year bracket being the second highest with 33 % (Table 4).

**Figure:**
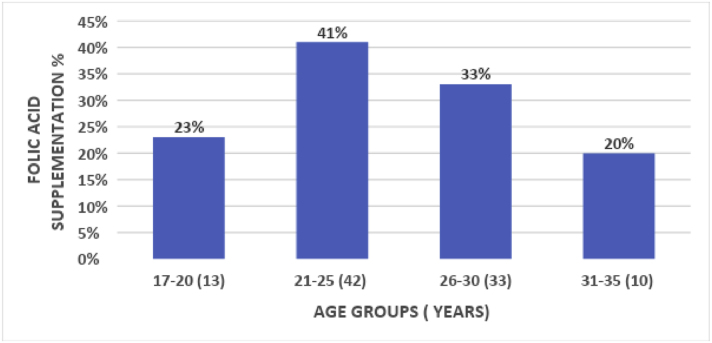

**Table 4:** Percentage between age groups and folic acid supplementation.

		Medication since the time of conception (folic acid)		Total	Chi-square value	p-value
		folic acid	no med			
Age of conceiving (in years)	17–20	6	18	24	1.23	0.746
	21–25	13	36	49		
	26–30	8	13	21		
	31–35	2	4	6		
Total		29	71	100		

Among the 11, 5 women were consuming other vitamin supplements.

An analysis of the timing of visits and knowledge of folic acid's benefits during pregnancy showed that only 11 women recognized its advantages. Awareness decreased in the third trimester, with fewer women informed compared to those in the first trimester (Table 5).

**Figure:**
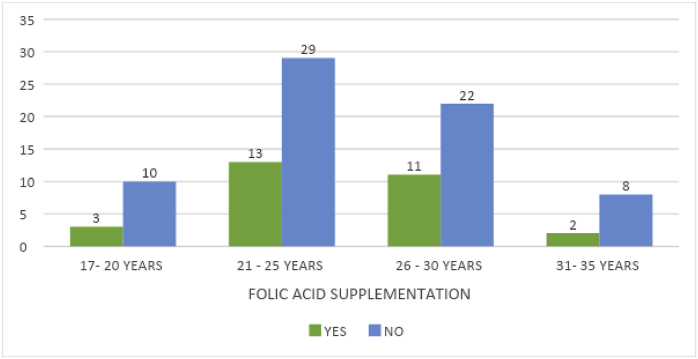

**Table 5:** Association between duration of medication and benefits of folic acid.

		benefits of folic acid		Total	Chi-square value	p-value
		no	yes			
medication durations (in months)	10.	2	0	2	16.963	**.049**
	2.	9	5	14		
	3.	2	1	3		
	4.	2	0	2		
	5.	2	1	3		
	6.	12	2	14		
	7.	3	0	3		
	8.	23	1	24		
	9.	10	1	11		
	no med	24	0	24		
Total		89	11	100		

The chi-square test demonstrated a statistically significant association between medication duration and folic acid benefits. As per the age of conceiving, consumption of folic acid was found to be the lowest among the 31–35-year-olds, followed by the 17–20 age group. A near-maximum usage of folic acid was reported in the age groups of 21–25 and 26–30 years.

A total of 42 mothers in the age group of 21–25 years, shows the mean age of mothers getting pregnant. Of these, 29 mothers comprising 70 % are not consuming folic acid.

The total number of consanguineal marriages is 44 with only 12 subjects taking folic acid whereas the non-consanguineal married mothers are 56 in number of which only 17 are taking folic acid. This highlights the fact that women in consanguineous marriages are at a higher risk for orofacial clefts with the genetic factor involved.

## 4 Discussion

An anomaly of cleft lip and palate relates primarily to an environmental factor arising due to folate deficiency. In addition to compelling evidence on other environmental determinants of cleft lip and palate in a population. All these factors are connected with maternal welfare and prenatal exposures such as gestational hypertension, gestational diabetes, seizures, and other medically compromised conditions. Additionally, these factors may show different geographical prevalence rates due to ethnicity, cultural practices, or socio-economic status.

Together, genetic vulnerability interacting with different regulatory mechanisms and exposure to the environment explain why some populations are more prone than others globally to cleft lip and palate.

Folic acid (pteroylmonoglutamic acid) has multifactorial benefits and several studies have substantiated its role in maintaining the body's physiological equilibrium (see table).

A comprehensive knowledge of the biochemistry of folic acid synthesis is essential to elicit its role in nutritional and reproductive biology as illustrated in the flow chart.

**Figure:**
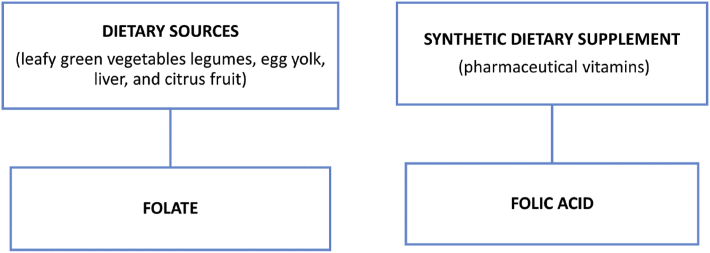

To become metabolically active, folic acid is converted to THF and L- METHYLFOLATE which includes the following enzymes DHF reductase (DHFR) and MTHFR that play a pivotal role in all biological processes related to the metabolism of folate and methionine.

**Fig. 1:**
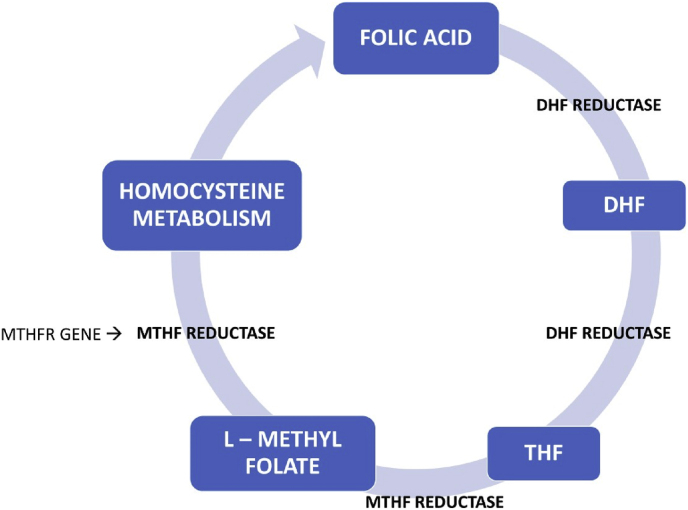
Metabolic pathway of folic acid.

This conversion is essential to produce L-methyl folate for facilitating the one-carbon transfer reactions (methyl donations) required for purine/pyrimidine synthesis in the course of DNA and RNA assembly, DNA methylation, and the regulation of homocysteine metabolism (see Fig. 1).

A daily dietary intake requirement of 600 mcg of folic acid is essential to support the optimal growth and development of the fetus. This recommendation is crucial to ensure the proper nourishment of the baby during pregnancy.

When diagnosing folic acid levels, it is recommended to conduct initial laboratory tests such as a complete blood count (CBC) and a peripheral smear (PS). Additionally, ordering serum vitamin B12 and folate levels can be beneficial in distinguishing between the two nutrients.

Deficiency in serum folate levels is classified as <2 ng/mL, while levels >4 ng/mL are considered within the normal range. For levels falling between 2 and 4 ng/mL, further confirmation is advised through the measurement of methylmalonic acid (MMA) and homocysteine levels revealing anemia, manifesting as a decrease in hemoglobin and hematocrit levels.^3^

Thus, patients being evaluated for folic acid deficiency should also be evaluated for vitamin B12 deficiency as both cause macrocytic anemia.

The 2015 guidelines by the International Federation of Obstetrics and Gynecology (FIGO) are referenced, emphasizing the need for increased folic acid intake before conception and during organogenesis to reduce the risk of adverse fetal outcomes. The paper provides an update on folic acid supplementation for women planning pregnancy or reproductive age to prevent neural tube defects (NTDs) and other congenital malformations. Women should supplement with 0.4–1.0 mg of folic acid daily before conception, in addition to dietary fortification. Also highlights the importance of higher folic acid doses for specific groups like those with previous NTD pregnancies, low folic acid intake, genetic variations, exposure to antifolate medications, smokers, diabetics, and obese individuals.^8^

Due to the effect of folate deficiency on neural tube formation, fortification of grains with folic acid has been mandated in many countries.

Folic acid-fortified iodized salt consumption led to a significant increase in serum folate levels among women of reproductive age in rural India, potentially preventing serious birth defects like spina bifida and anencephaly. There was a 3.7-fold increase in median serum folate levels among participants after they consumed approximately 300 μg/d of folic acid provided by the study salt^.^^9^

Consumption of fortified salt significantly increased median serum folate concentrations The article concluded by suggesting the implementation of folic acid fortification programs in public health policies. Another 3-month study of women of reproductive age consuming 100-μg and 400-μg folic acid supplement pills daily reported serum folate levels increase by 2.0-fold and 3.8-fold, respectively.^9^

The Indian recommendations for daily supplementation of multivitamins should begin at least 3 months before conception and continue until 10–12 weeks post-conception is 5 mg and 12 weeks post-conception continuing throughout pregnancy and the postpartum period (4–6 weeks or as long as breastfeeding continues) is 0.4–1.0 mg.^10^

The National Anemia Prophylaxis Program in India mandates folic acid and iron (0.5 and 60 mg) supplementation to young and pregnant girls.

In our survey assessment among a group of 52 individuals, it was observed that 24 subjects across all trimesters are not utilizing any form of supplementation and solely depend on dietary sources for their folate intake.

Indian Council of Medical Research recommends 200 μg as the intake of dietary folate for adult males and females. An additional requirement of 300 and 100 μg, respectively, during pregnancy and lactation was decided to be added to meet the factorial extra needs.^11^

Folic acid can be obtained from multivitamins, prenatal vitamins, B-complex vitamin supplements, and folic acid-only supplements.

Approximately 85 % of supplemental folic acid combined on an empty stomach is absorbed when taken alongside food, while nearly 100 % of absorption is achieved when taken without food.

There are also dietary supplements available containing 5-MTHF, which may be more advantageous for certain individuals compared to folic acid supplementation. The bioavailability of 5-MTHF in supplements is either equal to or greater than that of folic acid. However, formal conversion factors between mcg and mcg DFE for 5-MTHF have not been officially established.

Among Indian families, there is a proven incurrence of genetic abnormalities leading to high prevalence in first-degree consanguineous marriages^.^^12^

The first-trimester screening is crucial in genetic mapping and identification of the causative gene. IRF6 may be one such gene associated with non-syndromic orofacial clefts.^13^ Other such tests like Genotype tests, and carrier screening are also advised.

## 5 Conclusion

The study concludes that folic acid is crucial for various body functions, particularly in pregnant women, and emphasizes the need for increased awareness regarding its benefits and proper usage at all levels. It highlights a significant lack of knowledge among pregnant women about folic acid, with only 11 out of 100 participants aware of its nutritional significance. The research indicates that deficiencies in folic acid can be prevented by addressing underlying causes and stresses the importance of supplementation during the early stages of gestation to prevent orofacial clefts and promote overall well-being. Future deficiencies can be prevented by identifying and addressing an underlying cause of the folic acid deficiency.^6^

## 6 Strengths

1 The study utilized a randomized design with purposive sampling, which enhances the reliability of the findings by ensuring a diverse representation of pregnant women attending a specific hospital
2 A comprehensive questionnaire was developed, consisting of 13 questions that covered various aspects of folic acid awareness and its perceived benefits, allowing for a thorough assessment of participants' knowledge.
3 .The study highlighted a significant gap in awareness regarding folic acid, with only 11 % of participants informed about its benefits, emphasizing the need for targeted educational interventions
4 The data collected over nine months provided a substantial timeframe for observing trends in awareness and supplementation practices among pregnant women.
5. The focus on the first and second trimesters as critical periods for folic acid intake underscores the importance of timing in maternal nutrition, which is vital for embryogenesis.
6 Limited studies on the awareness of folic acid either as a fortified or nutritional supplementation.

## 7 Limitations

1. The study involved a small sample of just 100 participants, which may not accurately reflect the larger population of pregnant women and could limit the generalizability of the results.
2. The survey depended on self-reported information, leading to potential biases and inaccuracies in answers related to folic acid awareness and supplementation.
3. The research did not consider the educational background and income levels of participants, factors that could affect their understanding and access to folic acid supplementation.
4. The questionnaire was administered within a specific timeframe (August 2023–April 2024), which might not capture seasonal changes in health behaviours or access to healthcare services.
5. There was no follow-up to evaluate the long-term effects of folic acid awareness and supplementation on the health outcomes of mothers and their babies.

**Table:** 

NAME	CONSENT
Kushbu	Agree
Parvathi	Agree
Manjula	Agree
Geethalakshmi	Agree
Amsaveni	Agree
Ramya	Agree
Meena	Agree
Iswarya	Agree
Nisha	Agree
Harini	Agree
Smitha	Agree
Sundari	Agree
Malarkodi	Agree
Selvi	Agree
Sudha	Agree
Swathi	Agree
Sumitra	Agree
Tamilselvi	Agree
Ananthi	Agree
Sorna	Agree
Sankari	Agree
Puspha	Agree
Aananthi	Agree
Kala	Agree
Vaishnavi	Agree
Prasana	Agree
Shobana	Agree
Vanaja	Agree
Renuka	Agree
Priya bharathi	Agree
Logamal	Agree
Muthulakshmi	Agree
Amudha	Agree
Shalini	Agree
Selvi	Agree
Krishnaveni	Agree
Chandralekha	Agree
Tamilmalar	Agree
Varalaksmi	Agree
Vani	Agree
Ramya	Agree
Ragavi	Agree
Rajeswari	Agree
Banu	Agree
Sathiya pooja	Agree
Shalini	Agree
Malaiarasi	Agree
Sheela	Agree
Gomathy	Agree
Archana	Agree
Pachaiammal	Agree
Gauthami	Agree
Kanmani	Agree
Vasantha	Agree
Shella	Agree
Priyanka	Agree
Shobana	Agree
Mullai	Agree
Vennila	Agree
Nandhidha	Agree
Laxshmi	Agree
Chitra	Agree
Parameshwari	Agree
Suji	Agree
Nandhinni	Agree
Vasanthi	Agree
Ashwini	Agree
Krishnammal	Agree
Sumathi	Agree
Priya	Agree
Nithya	Agree
Yamini	Agree
Nagalaxshimi	Agree
Sugandhi	Agree
Rani	Agree
Manimekalai	Agree
Fathima	Agree
Mary	Agree
Purani	Agree
Mythili	Agree
Brindha	Agree
Maragadham	Agree
Jothylakshmi	Agree
Suriyakumari	Agree
Valli	Agree
Vijaya	Agree
Kamachi	Agree
Subulakshmi	Agree
Ammu	Agree
Laxshmi	Agree
Sundharivalli	Agree
Sivasakthi	Agree
Ashwini	Agree
Kala	Agree
Anapurani	Agree
Nagavalli	Agree
Magarakulazhi	Agree
Chembarathi	Agree
Visalachi	Agree
Kalliammal	Agree

## Source of funding

There has been no involvement in the source of funding for the conduct of the research and/or preparation of the article.

## Declaration of competing interest

Declaration of competing interest: The authors declare that they have no known competing financial interests or personal relationships that could have appeared to influence the work reported in this paper.
